# Associations between *LMO1* gene polymorphisms and Wilms' tumor susceptibility

**DOI:** 10.18632/oncotarget.16926

**Published:** 2017-04-07

**Authors:** Guo-Chang Liu, Zhen-Jian Zhuo, Shi-Bo Zhu, Jinhong Zhu, Wei Jia, Zhang Zhao, Jin-Hua Hu, Jing He, Feng-Hua Wang, Wen Fu

**Affiliations:** ^1^ Department of Pediatric Urology, Department of Pediatric Surgery, Guangzhou Institute of Pediatrics, Guangzhou Women and Children's Medical Center, Guangzhou Medical University, Guangzhou 510623, Guangdong, China; ^2^ School of Chinese Medicine, Faculty of Medicine, The Chinese University of Hong Kong, Hong Kong 999077, China; ^3^ Molecular Epidemiology Laboratory and Department of Laboratory Medicine, Harbin Medical University Cancer Hospital, Harbin 150040, Heilongjiang, China

**Keywords:** LMO1, Wilms’ tumor, polymorphism, susceptibility, GWAS

## Abstract

Wilms’ tumor is the most common childhood renal malignancy. A genome-wide association study identified LIM domain only 1 (LMO1) as having oncogenic potential. We examined the associations between *LMO1* gene polymorphisms and susceptibility to Wilms’ tumor. In this hospital-based, case-control study, we recruited 145 children with Wilms’ tumor and 531 cancer-free children. Four polymorphisms (rs110419 A>G, rs4758051 G>A, rs10840002 A>G and rs204938 A>G) were genotyped using Taqman methodology. Odds ratios (ORs) and 95% confidence intervals (CIs) were calculated to measure the associations between selected polymorphisms and Wilms’ tumor susceptibility. Only rs110419 AG was found to be protective against Wilms’ tumor (adjusted OR = 0.62, 95% CI = 0.41–0.94, *P* = 0.024) when compared to rs110419 AA. Wilms’ tumor risk was markedly greater in children with 1–4 risk genotypes (nucleotide alterations) than in those with no risk genotypes (adjusted OR = 1.84, 95% CI = 1.25–2.69, *P* = 0.002). In a stratified analysis, the protective effect of rs110419 AG/GG was predominant in males. The association of 1–4 risk genotypes with Wilms’ tumor risk was limited to subgroups of children who were >18 months old, female, and in clinical stages III+IV. Thus, *LMO1* gene polymorphisms may contribute to Wilms’ tumor risk, but this conclusion should be validated in other populations and larger studies.

## INTRODUCTION

Wilms’ tumor, also known as nephroblastoma, is the most common renal malignancy in children [1, 2]. The incidence of Wilms’ tumor is about 1 in 10,000 children of Western descent < 15 years of age [3]. Wilms’ tumor is less prevalent in China than in Western countries, with an incidence of ~3.3 per million [4]. Dramatic progress has been made in the treatment of children with Wilms’ tumor, with overall survival rates exceeding 90% in 2009, compared with about 30% in the 1930s [5, 6]. This success has mainly been due to multidisciplinary therapy and multi-institutional clinical trials [7, 8]. However, about 25% of affected children cannot be cured by current treatments, and approximately 50% of these children will die of Wilms’ tumor despite aggressive re-treatment [9, 10].

Wilms’ tumor appears to arise from nephrogenic rests, lesions that form when mesenchymal tissue fails to differentiate to nephrons [11]. Although there have been major advances in understanding the pathogenesis of Wilms’ tumor, the molecular mechanisms responsible for this differentiation failure are not completely understood. Chromosomal abnormalities are known to promote the formation of Wilms’ tumor by stimulating the uncontrolled growth of these undifferentiated cells [12, 13]. While a substantial proportion of Wilms’ tumor cases are sporadic and unilateral, 1–2% are hereditary [14–16]. Thus, genetic factors may also be involved in the predisposition to and aggressiveness of Wilms’ tumor [17, 18].

The Wilms’ tumor gene was the first identified suppressor of Wilms’ tumor development [19]. Thereafter, several susceptibility genes were found predispose individuals to Wilms’ tumor, such as *FWT1* [20], *FWT2* [21], *BRCA2* [22], *TP53* [23, 24], *BARD1* [25] and *CTR9* [26]. The LIM domain only 1 (*LMO1*) gene is located at 11p15, and encodes a cysteine-rich two-LIM-domain transcriptional regulator. *LMO1*, along with three paralogues (*LMO2*, *LMO3* and *LMO4*), is a member of the *LMO* gene superfamily. *LMO1* is abundantly expressed in the nervous system and has been implicated in its development [27]. Overexpression of LMO1 was initially found in patients with T-cell acute lymphoblastic leukemia [28]. Although numerous subsequent studies have demonstrated the association of this critical gene with neuroblastoma risk [29–31], none have investigated the associations between *LMO1* single nucleotide polymorphisms (SNPs) and Wilms’ tumor risk.

Four polymorphisms in *LMO1* (rs110419 A>G, rs4758051 G>A, rs10840002 A>G and rs204938 A>G) were found to be associated with the risk of several cancers in a genome-wide association study (GWAS) [29, 32]. We speculated that these polymorphisms might also contribute to the risk of Wilms’ tumor. Thus, we examined the associations between these *LMO1* polymorphisms and Wilms’ tumor risk in Southern Chinese children.

## RESULTS

### Population characteristics

In total, 145 Wilms’ tumor patients and 531 cancer-free controls were included in our analysis. Their demographic characteristics are presented in Supplementary Table 1. The mean age was 26.17 ± 21.48 months for the Wilms’ tumor patients and 29.73 ± 24.86 months for controls. The distributions of age (*P* = 0.725) and gender (*P* = 0.956) did not differ significantly between the cases and controls. Regarding the clinical stages of the cases, 4 (2.76%), 49 (33.79%), 50 (34.48%), 33 (22.76%), and 9 (6.21%) cases were classified into stages I-IV and ‘not available’, respectively, in accordance with National Wilms Tumor Study-5 criteria [33].

### Associations between *LMO1* gene polymorphisms and Wilms’ tumor risk

We then genotyped the Wilms’ tumor patients and cancer-free controls for four *LMO1* gene polymorphisms (rs110419 A>G, rs4758051 G>A, rs10840002 A>G and rs204938 A>G). The *LMO1* genotype frequencies and their associations with Wilms’ tumor risk are listed in Table 1. The observed genotype frequencies among the controls were all in agreement with Hardy-Weinberg equilibrium. Among the four polymorphisms, only rs1140419 A>G was associated with Wilms’ tumor risk – the risk was lower for children with the AG genotype than for those with the AA genotype (adjusted odds ratio [OR] = 0.62, 95% confidence interval [CI] = 0.41–0.94, *P* = 0.024). We further examined the joint effect of these risk genotypes on Wilms’ tumor susceptibility. The risk for developing Wilms’ tumor was significantly greater in individuals carrying one to four risk genotypes (nucleotide alterations) than in those with no risk genotypes (adjusted OR = 1.84, 95% CI = 1.25–2.69, *P* = 0.002).

**Table 1:** Associations between *LMO1* gene polymorphisms and Wilms’ tumor susceptibility

Genotype	Cases (*N* = 143)	Controls (*N* = 531)	*P*^a^	Crude OR (95% CI)	*P*	Adjusted OR (95% CI) ^b^	*P*^b^
rs110419 (HWE = 0.248)							
AA	55 (38.46)	159 (29.94)		1.00		1.00	
AG	59 (41.26)	275 (51.79)		**0.62 (0.41–0.94)**	**0.024**	**0.62 (0.41–0.94)**	0.024
GG	29 (20.28)	97 (18.27)		0.86 (0.52–1.45)	0.579	0.87 (0.52–1.46)	0.605
Additive			0.070	0.87 (0.67–1.14)	0.323	0.88 (0.67–1.15)	0.335
Dominant	88 (61.54)	372 (70.06)	0.055	0.68 (0.47–1.01)	0.053	0.68 (0.47–1.01)	0.053
Recessive	114 (81.73)	434 (81.73)	0.587	1.14 (0.72–1.81)	0.584	1.15 (0.72–1.83)	0.554
rs4758051 (HWE = 0.199)							
GG	52 (36.36)	194 (36.53)		1.00		1.00	
AG	64 (44.76)	242 (45.57)		0.99 (0.65–1.49)	0.949	0.98 (0.65–1.49)	0.936
AA	27 (18.88)	95 (17.89)		1.06 (0.63–1.79)	0.827	1.05 (0.62–1.77)	0.863
Additive			0.962	1.02 (0.79–1.32)	0.863	1.02 (0.79–1.32)	0.898
Dominant	91 (63.64)	337 (63.47)	0.970	1.01 (0.69–1.48)	0.970	1.00 (0.68–1.47)	0.995
Recessive	116 (81.12)	436 (82.11)	0.786	1.07 (0.67–1.72)	0.785	1.06 (0.66–1.70)	0.818
rs10840002 (HWE = 0.070)							
AA	46 (32.17)	182 (34.27)		1.00		1.00	
AG	62 (43.36)	240 (45.20)		1.02 (0.67–1.57)	0.920	1.02 (0.67–1.57)	0.929
GG	35 (24.48)	109 (20.53)		1.27 (0.77–2.09)	0.348	1.26 (0.77–2.08)	0.362
Additive			0.597	1.12 (0.87–1.44)	0.381	1.12 (0.87–1.44)	0.395
Dominant	97 (67.83)	349 (65.73)	0.635	1.10 (0.74–1.63)	0.637	1.10 (0.74–1.63)	0.650
Recessive	108 (75.52)	422 (79.47)	0.312	1.26 (0.81–1.94)	0.307	1.25 (0.81–1.93)	0.319
rs204938 (HWE = 0.153)							
AA	94 (65.73)	354 (66.67)		1.00		1.00	
AG	42 (29.37)	165 (31.07)		0.96 (0.64–1.44)	0.839	0.96 (0.64–1.44)	0.830
GG	7 (4.90)	12 (2.26)		2.20 (0.84–5.73)	0.108	2.20 (0.84–5.75)	0.109
Additive			0.280	1.13 (0.81–1.58)	0.481	1.13 (0.80–1.58)	0.487
Dominant	49 (34.27)	177 (33.33)	0.834	1.04 (0.71–1.54)	0.833	1.04 (0.70–1.54)	0.842
Recessive	136 (95.10)	519 (97.74)	0.114	2.23 (0.86–5.76)	0.099	2.23 (0.86–5.78)	0.099
Combined effect of risk genotypes							
0	51 (35.66)	268 (50.47)		1.00		1.00	
1–4	92 (64.34)	263 (49.53)	0.002	**1.84 (1.25–2.69)**	**0.002**	**1.84 (1.25–2.69)**	0.002

OR: odds ratio; CI: confidence interval; HWE: Hardy–Weinberg equilibrium.

^a^χ^2^ test for genotype distributions between Wilms’ tumor patients and controls.

^b^Adjusted for age and gender.

### Stratification analysis

We further evaluated the relationship between the *LMO1* risk genotypes and Wilms’ tumor susceptibility in subjects stratified by age, gender, and clinical stage (Table 2). The stratification analysis indicated that the rs110419 AG/GG genotype was more likely to reduce Wilms’ tumor risk in males (crude OR = 0.60, 95% CI = 0.36–0.996, *P* = 0.048), but this association disappeared after adjustment for age and gender (adjusted OR = 0.61, 95% CI = 0.36–1.01, *P* = 0.057). No significant associations between rs110419 A>G and Wilms’ tumor risk were observed in the age or clinical-stage subgroups. The stratification analysis also indicated that the association of one to four risk genotypes with increased Wilms’ tumor risk was limited to the subjects who were >18 months old (adjusted OR = 2.69, 95% CI = 1.57–4.61, *P* = 0.0003), female (adjusted OR = 2.67, 95% CI = 1.47–4.85, *P* = 0.001), and in clinical stages III+IV (adjusted OR = 2.16, 95% CI = 1.31–3.55, *P* = 0.002).

**Table 2:** Stratification analysis of the associations between risk genotypes and Wilms’ tumor susceptibility

Variables	rs110419 (cases/controls)		OR	*P*	Adjusted OR^a^	*P*^a^	Risk genotypes (cases/controls)		OR	*P*	Adjusted OR a	*P*^a^
	AA	AG/GG	(95% CI)		(95% CI)		0	1–4	(95% CI)		(95% CI)	
Age, months												
≤18	24/74	41/159	0.80 (0.45–1.41)	0.433	0.80 (0.45–1.41)	0.434	28/110	37/123	1.18 (0.68–2.06)	0.555	1.17 (0.67–2.04)	0.575
>18	31/85	47/213	0.61 (0.36–1.02)	0.057	0.61 (0.36–1.02)	0.059	23/158	55/140	**2.70 (1.58–4.62)**	**0.0003**	**2.69 (1.57–4.61)**	**0.0003**
Gender												
Female	23/73	41/160	0.81 (0.46–1.45)	0.486	0.81 (0.45–1.44)	0.468	19/123	45/110	**2.65 (1.46–4.80)**	**0.001**	**2.67 (1.47–4.85)**	**0.001**
Male	32/86	47/212	**0.60 (0.36–0.996)**	**0.048**	0.61 (0.36–1.01)	0.057	32/145	47/153	1.39 (0.84–2.30)	0.198	1.37 (0.82–2.26)	0.227
Clinical stages												
I+II	22/159	31/372	0.60 (0.34–1.07)	0.085	0.61 (0.34–1.08)	0.091	23/268	30/263	1.33 (0.75–2.35)	0.327	1.31 (0.74–2.33)	0.358
III+IV	28/159	53/372	0.81 (0.49–1.33)	0.401	0.81 (0.49–1.32)	0.396	26/268	55/263	**2.16 (1.31–3.54)**	**0.002**	**2.16 (1.31–3.55)**	**0.002**

^a^Adjusted for age and gender.

OR, odds ratio. CI, confidence interval.

### Haplotype analysis and false-positive report probability (FPRP) analysis

The inferred haplotypes for the *LMO1* gene (in the order of rs110419, rs4758051, rs10840002 and rs204938) and their associations with Wilms’ tumor risk are shown in Table 3. Wilms’ tumor risk was greater in GGAG haplotype carriers (OR = 3.23, 95% CI = 1.26–8.26, *P* = 0.014) than in GGAA haplotype carriers. Likewise, the GGGA haplotype was also associated with greater Wilms’ tumor risk than GGAA (OR = 3.46, 95% CI = 1.46–8.18, *P* = 0.005).

**Table 3:** The frequencies of inferred *LMO1* gene haplotypes based on observed genotypes, and their associations with Wilms’ tumor susceptibility

Haplotypes^a^	Cases (*n* = 286)	Controls (*n* = 1062)	Crude OR (95% CI)	*P*	Adjusted OR^b^ (95% CI)	*P*^b^
GGAA	53 (18.53)	276 (25.99)	1.00		1.00	
GGAG	8 (2.80)	12 (1.13)	**3.23 (1.26–8.26)**	**0.014**	**3.23 (1.26–8.28)**	**0.015**
GGGA	10 (3.50)	14 (1.32)	**3.46 (1.46–8.18)**	**0.005**	**3.53 (1.49–8.35)**	**0.004**
GAAA	2 (0.70)	0 (0.00)	/	/	/	/
GAGA	39 (13.64)	149 (14.03)	1.27 (0.81–1.99)	0.306	1.28 (0.81–2.01)	0.293
GAGG	5 (1.75)	18 (1.69)	1.35 (0.48–3.77)	0.573	1.35 (0.48–3.79)	0.570
AGAA	73 (25.52)	253 (23.82)	1.40 (0.95–2.06)	0.090	1.41 (0.96–2.07)	0.083
AGAG	18 (6.29)	60 (5.65)	1.45 (0.80–2.64)	0.222	1.45 (0.80–2.65)	0.223
AGGA	5 (1.75)	8 (0.75)	3.03 (0.96–9.59)	0.060	2.96 (0.93–9.43)	0.066
AGGG	1 (0.35)	7 (0.66)	0.69 (0.08–5.73)	0.733	0.72 (0.09–6.00)	0.762
AAAA	0 (0.00)	3 (0.28)	/	/	/	/
AAGA	48 (16.78)	170 (16.01)	1.37 (0.89–2.10)	0.153	1.36 (0.88–2.09)	0.164
AAGG	24 (8.39)	92 (8.66)	1.26 (0.74–2.15)	0.390	1.26 (0.74–2.15)	0.395

^a^The haplotype order is rs110419, rs4758051, rs10840002, rs204938.

^b^Obtained from logistic regression models adjusted for age and gender.

OR, odds ratio. CI, confidence interval.

In the FPRP analysis (Table 4), due to the small sample size, nearly all of the significant findings disappeared at a prior probability level of 0.1 and an FPRP threshold of 0.2, except for the increased Wilms’ tumor risk in carriers of one to four risk genotypes (FPRP = 0.099).

**Table 4:** False-positive report probability values for the significant findings

Genotype	Crude OR (95% CI)	*P*^a^	Statistical power^b^	Prior probability				
				0.25	0.1	0.01	0.001	0.0001
*LMO1* rs110419 A>G								
AG vs. AA	0.62 (0.41–0.94)	0.024	0.441	0.140	0.329	0.844	0.982	0.998
AG/GG vs. AA								
Males	0.60 (0.36–0.996)	0.048	0.328	0.305	0.568	0.935	0.993	0.999
*Risk genotypes*								
1–4 vs. 0	1.84 (1.25–2.69)	0.002	0.165	0.035	0.099	0.546	0.924	0.992
>18 months	2.70 (1.58–4.62)	0.0003	0.008	0.107	0.264	0.798	0.976	0.998
Females	2.65 (1.46–4.80)	0.001	0.015	0.164	0.371	0.867	0.985	0.998
Stage III+IV	2.16 (1.31–3.54)	0.002	0.038	0.138	0.324	0.841	0.982	0.998
*Haplotypes*								
GGAG vs. GGAA	3.23 (1.26–8.26)	0.014	0.065	0.400	0.667	0.957	0.996	1.000
GGGA vs. GGAA	3.46 (1.46–8.18)	0.005	0.036	0.284	0.543	0.929	0.992	0.999

^a^A χ*2* test was used to calculate the genotype frequency distributions.

^b^Statistical power was calculated from the number of observations in the subgroup and the ORs and *P* values in this table.

OR, odds ratio. CI, confidence interval.

## DISCUSSION

In the present hospital-based case-control study of 145 children with Wilms’ tumor and 531 cancer-free controls, we investigated the associations of four GWAS-identified *LMO1* gene polymorphisms with Wilms’ tumor susceptibility. We discovered that rs110419 A>G was associated with Wilms’ tumor susceptibility in a Southern Chinese population. To the best of our knowledge, this is the first report of an association between a *LMO1* gene polymorphism and Wilms’ tumor susceptibility in Chinese children.

There is overwhelming evidence that *LMO1* is a critical determinant of cancer susceptibility. In a GWAS conducted among individuals of European descent, Wang et al. discovered that four genetic variants of *LMO1* (rs110419 A>G, rs4758051 G>A, rs10840002 A>G and rs204938 A>G) contributed to the tumorigenesis of neuroblastoma [29]. Subsequently, this relationship was confirmed in four other epidemiological studies among people of different ethnicities [30, 32, 34, 35]. Beuten et al. identified an association between another genetic variant (rs442264 A>G) in the *LMO1* gene and acute lymphoblastic leukemia susceptibility in a population of Caucasian children (163 cases and 251 controls) [36]. Recently, Oldridge et al. found that the rs2168101 G>T polymorphism in *LMO1* predisposed individuals to neuroblastoma. The authors also performed biological function studies to elucidate the oncogenic role of this polymorphism in tumor cells [37].

Despite the growing body of research demonstrating the associations of *LMO1* gene variants with cancer susceptibility, until now, no study had investigated the relationship between *LMO1* polymorphisms and Wilms’ tumor risk. Here, we performed an epidemiologic study on the associations between four *LMO1* gene polymorphisms and Wilms’ tumor risk in 145 affected children and 531 healthy children. We found that the rs110419 AG genotype reduced Wilms’ tumor risk in the overall analysis, while we did not detect significant associations between the other three polymorphisms and Wilms’ tumor risk. However, we found that the predisposition to Wilms’ tumor was significantly greater in children with one to four risk genotypes than in those with no risk genotypes. This relationship was significant in children who were > 18 months old, female, and in clinical stages III+IV, but not in their counterpart subgroups. The above conflicting results may be ascribed to the following: 1) the relatively small sample size, 2) the relatively weak impact of *LMO1* SNPs, and 3) the influence of environmental factors on Wilms’ tumor susceptibility.

Our study was the first to investigate the associations of *LMO1* gene polymorphisms with Wilms’ tumor risk in a Chinese population. However, several limitations should be considered in the interpretation of our results. Firstly, only 145 patients and 531 controls were included in this analysis. This relatively small sample size inevitably reduced the statistical power, especially for the stratification and FPRP analyses. Secondly, the inherent selection bias could not be completely eliminated, since our study was a hospital-based study with subjects restricted to South China. Thirdly, due to the nature of retrospective studies, some valuable information could not be collected, such as parental exposures and dietary intakes, which diminished the precision of the results. Finally, these four SNPs were identified in a GWAS on neuroblastoma, while the present study dealt with Wilms’ tumor. A GWAS regarding *LMO1* gene SNPs and Wilms’ tumor remains to be performed.

In conclusion, we determined that the rs110419 AG polymorphism in *LMO1* may reduce the susceptibility to Wilms’ tumor in a Southern Chinese population. Well-designed studies with larger sample sizes in different ethnicities should be performed in the future. Furthermore, other *LMO1* gene variants and gene-environment interactions should be investigated to provide essential insights into the etiology of Wilms’ tumor.

## MATERIALS AND METHODS

### Study subjects

Details on the recruited control subjects were reported previously [38–42]. For the present study, 145 patients with newly diagnosed and histologically confirmed Wilms’ tumor were recruited from the Department of Pediatric Urology, Guangzhou Women and Children's Medical Center between March 2001 and June 2016, while 531 cancer-free children undergoing routine physical examinations in the same hospital were randomly selected as controls. All the subjects were genetically unrelated ethnic Han Chinese from South China [24, 25, 43]. The response rate was approximately 90% for Wilms’ tumor patients and 95% for cancer-free controls. The current study was approved by the Institutional Review Board of Guangzhou Women and Children's Medical Center. Written informed consent was obtained from each participant's parents or legal guardians.

### Genotyping

About 2 mL of peripheral blood was collected from each subject for genotyping. Four *LMO1* gene SNPs (rs110419 A>G, rs4758051 G>A, rs10840002 A>G and rs204938 A>G) identified in a GWAS on neuroblastoma were chosen for genotyping [29]. Genomic DNA was isolated from peripheral blood leukocytes with a TIANamp Blood DNA Kit (TianGen Biotech, Beijing, China) [38, 40]. A 7900 Sequence Detection System (Applied Biosystems, Foster City, CA) and Taqman real-time PCR were used to genotype the *LMO1* SNPs, as described thoroughly elsewhere [44, 45]. To obtain convincing results, we performed the genotyping blindly, not knowing whether each subject was a case or control. We also randomly selected 10% of the samples for repeated genotyping, and the genotype concordance was 100%.

### Statistical analysis

Hardy-Weinberg equilibrium was calculated with a goodness-of-fit χ^2^ test for the genotype frequencies in controls. A two-sided χ^2^ test was used to evaluate the differences in demographic variables and genotype frequencies between cases and controls. To estimate the associations between *LMO1* polymorphisms and Wilms’ tumor susceptibility, we calculated ORs and 95% CIs using unconditional logistic regression with adjustment for age and gender. We also assessed the associations of the various haplotypes with Wilms’ tumor susceptibility [46]. FPRP analysis was performed as described previously [47, 48]. *P* < 0.05 was considered statistically significant. All statistical analyses were performed with SAS software (Version 9.4; SAS Institute, Cary, NC).

## SUPPLEMENTARY MATERIALS FIGURES AND TABLES


