# Supplementary material for: Associations between LMO1 gene polymorphisms and Wilms' tumor susceptibility
Source: Oncotarget. 2017 Apr 7;8(31):50665–72. doi: 10.18632/oncotarget.16926 (PMC5584185; doi:10.18632/oncotarget.16926)
Supplement: Supplementary file 1 [file oncotarget-08-50665-s001.pdf]

Associations between *LMO1* gene polymorphisms and Wilms' tumor susceptibility

Supplementary Materials

Supplementary Table 1: Frequency distribution of selected variables in Wilms' tumor cases and cancer-free controls

Variables	Cases (n = 145)		Controls (n = 531)		P^a
	No.	%	No.	%	
Age, months					
Range	1~132		0.07~156		0.725
Mean ± SD	26.17 ± 21.48		29.73 ± 24.86		
≤ 18	66	45.52	233	43.88	
> 18	79	54.48	298	56.12	
Gender					0.956
Female	64	44.14	233	43.88	
Male	81	55.86	298	56.12	
Clinical stage					
I	4	2.76			
II	49	33.79			
III	50	34.48			
IV	33	22.76			
NA	9	6.21			

[a]Two-sided χ^2 test for distributions between Wilms' tumor cases and cancer-free controls.
SD, standard deviation; NA, not available.
